# Morphological Evolution of an Intertidal Mudflat in Relation to Mangrove Growth: Implications for Future Erosion Control

**DOI:** 10.3390/life14060711

**Published:** 2024-05-30

**Authors:** Nguyen Tan Phong, Nguyen Bao Thuan, Le Tan Loi, Huynh Van Quoc

**Affiliations:** 1 Faculty of Environment and Labour Safety, Ton Duc Thang University, Ho Chi Minh City, Vietnam; 202810009@student.tdtu.edu.vn; 2 Faculty of Architecture—Construction and Environment, Nam Can Tho University, Can Tho City, Vietnam; ltloi@nctu.edu.vn (L.T.L.); hvquoc@nctu.edu.vn (H.V.Q.)

**Keywords:** fine-grained sediment, mangrove growth, sand accumulation, soil compaction, vegetation cover

## Abstract

There is limited information regarding the influence of mangrove growth on the morphological evolution of intertidal mudflats. In this study, Tan Phu Dong district, Tien Giang Province, Vietnam, was selected for investigating how mangrove growth influenced the morphological evolution of an intertidal mudflat. The authors analyzed a series of satellite images (from 1995 and 2022), calculated the enhanced vegetation index (EVI), and documented field visits and observations in pursuit of the objective of the study. The findings revealed that fine-grained sediment accumulated as unconsolidated substratum (US) in the first step of the morphological evolution of the intertidal mudflat, with sediment accumulation of 910 ha in 1995. The US provided favorable conditions for mangroves to grow, while mangrove growth helped compact the US into a compact substratum (CS) in addition to promoting continuous sediment accumulation, increased the vegetation cover of the island, and elevated the substrate density of the remaining areas. As a result, the US and CS decreased steadily between 1995 and 2020, from 910 ha in 1995 to 401 ha in 2020 and from 433 ha in 2005 to 111 ha in 2020, respectively. Meanwhile, the low-vegetation area (LVA), medium-vegetation area (MVA), and high vegetation area (HVA) gradually increased between 1995 and 2015, from 0 ha in 1995 to 104 ha in 2015, from 0 ha in 1995 to 96 ha in 2015, and from 0 ha in 1995 to 114 ha in 2015, respectively. However, the LVA decreased slightly between 2015 and 2020 due to significant sand accumulation, which significantly killed the mangrove trees. In contrast, the MVA and HVA steadily increased between 2015 and 2020, from 96 ha in 2015 to 116 ha in 2020 and from 114 ha in 2015 to 221 ha in 2020, respectively. In 2022, there was a steady increase in HVA (298 ha in 2022), although the date of the 2022 satellite retrieval was 28 January 2022. This study recommends that the technical design of the existing coastal protection works should be revised or adapted to take account of sediment accumulation as the first step in the morphological evolution of the examined intertidal mudflat, rather than mangrove growth.

## 1. Introduction

Intertidal mudflats are an integral part of coastal ecosystems [1,2] and are often found at the edges of tidal estuaries [3]. Mudflats can be intertidal sandflats [4], vegetated salt marshes [5,6,7,8,9], or tidal inlets [10]. They contribute to dissipating the energy of incident waves [11,12,13], support large numbers of invertebrates and fish [14,15], and assist in recycling organic matter and nutrients from both terrestrial and marine sources [16,17].

Sediment supply, local morphology, hydrodynamics (tidal cycles, river flow, wind-induced waves), and biodiversity activity shape the morphological evolution of intertidal mudflats [18,19]. The local sediment supply to coastal marshes is strongly linked to the intertidal sediment dynamics [20]. Sediment tends to be deposited in areas where large tidal ranges and minor wind waves dominate [21]. Most of the suspended sediment deposited on coastal marshes originates from wind- and wave-induced intertidal sediment resuspension in the very close vicinity (more than 130 m) of the seaward marsh margin [20]. The maximum sediment deposition depends on the critical shear stress for erosion and on the settling velocity, particularly under the influence of asymmetry in flood and ebb transport (increase with increased settling velocity and critical shear stress) and gross transport rates (decrease with increased settling velocity and critical shear stress). The deposition rates are strongly dependent on the cross-sectional profile and increase the concavity of the profile [22]. Abundant supplies of fine-grained fluvial sediment and resuspension and dominant onshore wind directions play a crucial role in mudflat accretion [23]. Cross-shore sedimentation reduces the accretion rates [24].

Tides, currents, wave periods and directions, alongshore water level gradients, and winds affect the profile of intertidal mudflats [25]. The tidal currents contribute to the morphological evolution of channel–shoal systems [26]. Extremely shallow water conditions have a key influence on intertidal flat hydrodynamics and sediment dynamics [27] and play a critical role in determining the formation and evolution of micro-topographies on tidal flats [28]. A constant topographic elevation increases desiccation and consolidation processes that facilitate the transition of bare mud surfaces into ideal substrates where mangroves settle [29,30,31]. Inhomogeneous distributions of hydrodynamic forcing processes (including the underexplored role of wind), linear proportionality between bed level dynamics, and the local bed slope have spatiotemporal effects on bed level dynamics, particularly under the influence of storms [32]. In small (approximately 2 km) and sheltered tidal flats, waves cause the largest sediment resuspension when the water levels are near the mean sea level [33]. In extensive tidal flats (approximately 20 km) or in flats exposed to waves propagating from deep water, the waves also cause substrate erosion during high tides or large storm surges. The convexity, or the concavity, of the cross-shore profile of intertidal flats determines the dominant mode of morphologic variability [34]. Cross-shore and alongshore tidal flow, wave action, sediment transport, and changes in bed level determine the morpho-dynamics of intertidal mudflats [35].

Water turbidity and bio-stabilization play crucial roles in controlling the evolution of intertidal mudflat mass [36,37]. Biophysical stress divergence strongly influences the spatial self-organization on intertidal mudflats [38]. Sediment particulate organic matter (i.e., organic carbon (OC) and nitrogen (N) concentrations) interact with the evolution of estuarine intertidal mudflats [39].

Mangrove forests grow in areas ranging from intertidal habitats to riverine environments in the tropics and subtropics [40]. These forests naturally grow on intertidal mudflats [31,41,42] or stable areas [43,44,45,46,47,48] and do not enhance sedimentation [49]. In summary, all driving factors, morphological and coastal dynamics, of the morphological evolution of intertidal mudflats have been identified and analyzed according to their roles and influences.

Shorelines change due to the overexploitation of relatively fragile ecosystems on muddy coasts [50] and the negative effects of sea level rise [51]. Shoreline change causes land loss and damage to local livelihoods [52]. Shoreline change is normally controlled using engineered solutions [53,54], ecological engineering solutions [55], and land-use planning [56]. To date, engineered solutions involve the use of shoreline and offshore structures that aim primarily to dissipate the energy of strong incident waves [57,58]. However, engineered solutions pose threats to marine and coastal hydrodynamics [53,59]. Ecological engineering solutions involve the planting of mangrove seedlings, with additional protection provided by offshore structures [48]. The planting of mangrove seedlings does not guarantee success. Ecological engineering solutions have had limited success in controlling the shoreline change due to insufficient knowledge of coastal hydrodynamics [60]. The current land use policies are not practical in developing countries and do not result in sustainable coastal management [56].

Against this background, the crucial question of how mangrove growth influences the morphological evolution of intertidal mudflats remains unclear. This knowledge is important, particularly in contexts where mangrove forests are believed to trap sediments and contribute to the progradation and aggradation of deltas [61,62]. In addition, the existing coastal erosion control measures (ecological engineering solutions—mangrove transplantation—and engineered solutions—construction of shoreline and offshore protection structures) emphasize the utility of planting of mangrove seedlings in an attempt to trap sediment to establish intertidal mudflats and stabilize eroded coastal areas in Southeast Asia [63,64,65], including Vietnam [48,57,66,67,68,69].

We selected Con Ngang (hereby called Ngang Island), Tan Phu Dong district, Tien Giang Province, Vietnam, as an appropriate case study to address this question. Since 1995, Ngang Island has evolved from undergoing a constant fine-grained sediment accumulation and has experienced a strong natural growth of mangrove species. Moreover, other coastal areas in Tien Giang Province have been severely eroded. There is limited documentation on mangrove growth within the spatiotemporal evolution of Ngang Island. The Government of Vietnam emphasizes the protection of intertidal mudflats as a vital part of coastal protection measures for better adaptation to climate change and sea level rise in Vietnam [70]. Therefore, this study aimed to investigate the relationship between mangrove growth and the morphological evolution of intertidal mudflats in the Tan Phu Dong district, Tien Giang, Vietnam. Our goals were to document (1) how Ngang Island evolved morphologically between 1995 and 2022; (2) how mangrove forests grew during the evolution of Ngang Island. The first goal was achieved by analyzing the development of shoreline change between 1995 and 2022 using ArcGIS desktop 10.6 software and field visits and observations. The second one was achieved through the calculation of the enhanced vegetation index (EVI) and the compilation of detailed documentation during field visits. This study was implemented as a first step towards gaining a comprehensive understanding of the morphological evolution of Ngang Island. Thus, in this study, sediment was not sampled for sediment-size analysis, nor were soil profiles established for determining sediment accumulation volume and composition.

## 2. Materials and Methods

### 2.1. Site Description

Ngang Island is hydrodynamically linked to the coast of Tan Phu Dong district, Tien Giang Province, Vietnam. Ngang Island has a total area of approximately 16 km^2^. This island is predominantly affected by the southwest (May to October) and northeast (November to March) monsoon regimes. This island is strongly influenced by the East Sea tidal regime, which features semidiurnal tides and diurnal inequality. The tidal range varies between +1.8 and +2.2 m [61]. To date, much information is available related to the hydrodynamics and sedimentation conditions of the Tan Phu Dong district, which includes Ngang Island [66,67,68].

The bed level of Ngang Island varies between −3.0 m and −1.5 m in both seasons. The current speed is between 0.30 m/s and 0.35 m/s in both seasons. The total suspended sediment accumulations are between 0.2 m/s and 0.3 m/s.

**Figure 1:**
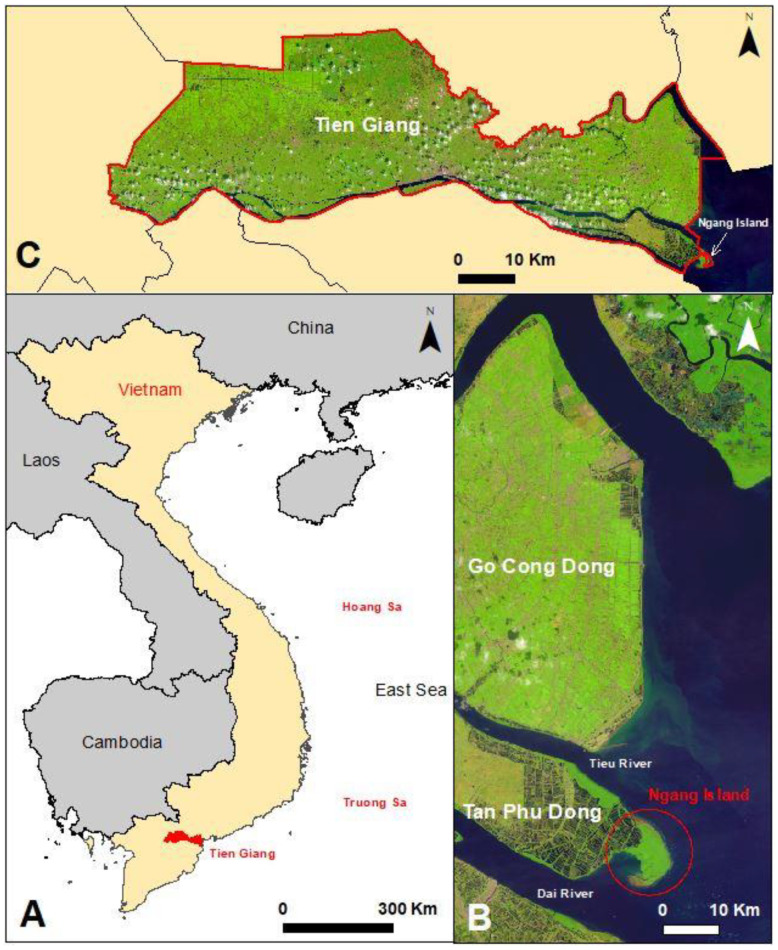
Location of Ngang Island. (**A**) The red dot shows the location of Tien Giang Province in Vietnam. (**B**) The shoreline of Tien Giang Province (Go Cong Dong and Tan Phu Dong districts). In (**B**), the red circle shows the location of Ngang Island. (**C**) The boundaries of Tien Giang Province (red line).

Ngang Island is predominantly influenced by the hydrodynamic regime of the Tieu River. The maximum velocity measured at the Tieu River mouth is 0.8 m/s at ebb tide and about 0.4–0.6 m/s at flood tide. The average wave height is greater than 0.64 m approximately 5 km off the shore, while the main direction of the incident waves is from the east. In contrast, the average wave height near the shore is less than 0.3 m during the southwest monsoon, and the main direction of the incoming waves is from the southeast [67]. The suspended sediment concentration (SSC) in the Tieu River ranges between 0.1 and 0.2 kg/m^3^ in the dry season and between 0.3 and 0.4 kg/m^3^ in the wet season [71] (Figure 1).

### 2.2. Methods

To commence the study, twelve satellite images (1995, 1997, 2000, 2004, 2005, 2008, 2010, 2013, 2015, 2018, 2020, and 2022) were retrieved from the United States Geological Survey (https://glovis.usgs.gov/ accessed on 18 December 2023)). The year 1995 was selected as the starting year for the analysis because Ngang Island began to exist in 1995. The 1997 satellite image was not used to establish the yearly shorelines but was analyzed to illustrate the evolution of Ngang Island using EVI values. The following years were crucial periods to gain insights into the morphological evolution of Ngang Island. The authors filtered these satellite images using two criteria, as follows: (1) selection of the images that covered the study area; (2) images having an average cloud cover of less than 20% (Table 1).

The satellite images were preprocessed prior to the shoreline analysis of Ngang Island and the EVI calculation. Top of atmospheric (TOA) reflectance was first determined in this study because this step helps remove elements that distort satellite images under different sun illumination conditions in accordance with previous recommendations [72,73]. Landsat 8 images were analyzed using the tools of ArcGIS desktop 10.6 software for calculating TOA reflectance, while Landsat 5 images were analyzed using the following formula:

$$R_{TOA}=\frac{\pi \times L}{1/d^{2}\times I_{sun}\times {cosӨ}_{Sun}}$$

where

R_TOA_ = top of atmosphere reflectance;

π = 3.14159;

L = L_λ_ (upward), radiance;

d = Earth–Sun distance in astronomical units;

I_Sun_ = mean extraterrestrial solar irradiance;

θ_Sun_ = solar zenith angle—the angle between the direction toward the Sun and the normal of the Earth’s surface.

Because Ngang Island is highly likely to be inundated by seasonal tides, satellite images from different dates were retrieved for analysis in this study. A shoreline is defined as a water–land boundary [74]. The authors strictly followed previous recommendations in developing the yearly water–land boundary before EVI methods and shoreline analysis were applied to avoid possible errors in calculating the levels of vegetation cover and shoreline change in Ngang Island. The yearly shorelines were established using the following steps: calculation of the modified normalized difference water index (mNDWI), calculation of the water frequency index (WFI), and establishment of yearly and water shapefiles. The mNDWI helps reduce and remove built-up land noise and showed excellent performance in extracting surface water [75]. The WFI helps highlight locations where water appears most frequently, before yearly land water shapefiles are established [76].

(1) The mNDWI formula is as follows:

$$mNDWI=\frac{Green-MIR}{Green+MIR}\;\;\;\;$$

where

Green = green band;

MIR = mid-infrared radiation.

The mNDWI value ranges between −1 and 1, and positive values represent pixels with water.

(2) The WFI formula is as follows:

$$WFI=\frac{N_{water}}{{N_{water}+N}_{land\;}}\;$$

where N_water_ and N_land_ denote the number of pixels that were observed as water and land within 1 year, respectively. By applying the WFI to the results of mNDWI, the authors determined the pixels associated with a high occurrence water from the water indices during a specific time, e.g., yearly or seasonally, and then extracted the representative surface water in the following section.

WFI pixel values greater than or equal to 0.5 (equivalent to a frequency of 50%) were reclassified as representative of the annual water/surface area.

(3) Because the island is ecologically and hydrologically linked to the Tan Phu Dong coast of Tien Giang Province, the authors decided to extract satellite images from multiple dates (1995, 1997, 2000, 2004, 2005, 2008, 2010, 2013, 2015, 2018, 2020, and 2022) as vector ‘shapefiles’ for both the Tan Phu Dong coast and the island.

In addition, overall accuracy (OA) and Kappa coefficient (KC) were used for evaluating the accuracy of the extraction results.

$$OA=\frac{TP+TN}{T}\times 100\%\;\;\;$$

where TP (True Positive) and TN (True Negative) represent, respectively, water and non-water pixels/points that match with the reference sites.

$$KC=\frac{\left(TS\times TCS\right)-Ʃ(Column\;Total\times Row\;Total)}{{TS}^{2}-Ʃ(Column\;Total-Row\;Total)}$$

where

TS = total sample;

TCS = total corrected sample.

The evaluation showed that the OA was 92%, and the KC was 0.90. These evaluation values showed that the proposed methods used in this study agreed with the previous recommendations that the maximum OA value be up to 100% and the maximum KC value be up to 1 [77,78].

The normalized difference vegetation index (NDVI) has been widely used as an indicator of vegetation health and degradation in mangrove forests [79,80,81,82] because the NDVI is a measure of the amount and vigor of vegetation on land surface. However, the NDVI is affected by soil brightness [83] and reaches saturation in high-biomass areas [84]. Therefore, the enhanced vegetation index (EVI) [85] is used as an alternative vegetation index to avoid possible errors caused by NDVI saturation and soil brightness because the EVI shows greater sensitivity to vegetation presence and reduces the atmospheric effects on the vegetation index values [85]. The EVI formula is as follows:

$$EVI=\frac{2.5\left(NIR-R\right)}{\left(NIR+2.4\times R+1.0\right)}$$

where

IR is the near-infrared band (Band 5—Landsat 8; Band 4—Landsat 7):

R is the red band (Band 4—Landsat 8; Band 3—Landsat 7).

**Figure 2:**
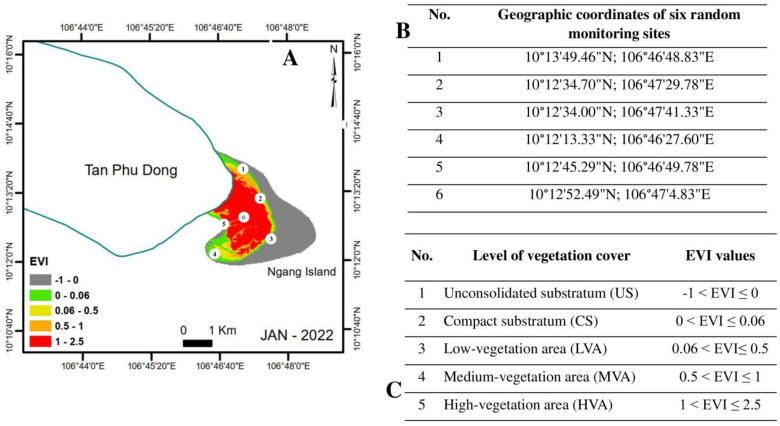
The random monitoring sites and levels of vegetation cover in Ngang Island, Tien Giang Province, Vietnam. (**A**) The 2022 EVI map with six random monitoring sites. (**B**) The geographic coordinates of six random monitoring sites. (**C**) The levels of vegetation cover and the corresponding EVI values.

The authors first classified Ngang Island surface into two classes, namely, vegetated area (VA) (mangrove forests) and non-vegetated area (NVA) (uncompacted fine-grained sediment and barren land). Six (6) field visits were organized to the site and its shoreline for ground truthing between January 2021 and December 2022. Ground truthing involved the collection of GPS data from six random monitoring sites, with brief descriptions of the examined areas and photographs of NVC and VC to determine the main geographical features, vegetation patterns, and recent occurrences for EVI analysis (the photographs taken during the field trips). The random monitoring sites were established in areas around and in the central area of Ngang Island to adequately illustrate the vegetation cover established over the entire period (Figure 2).

## 3. Results

### 3.1. Morphological Evolution of Ngang Island between 1995 and 2022

**Figure 3:**
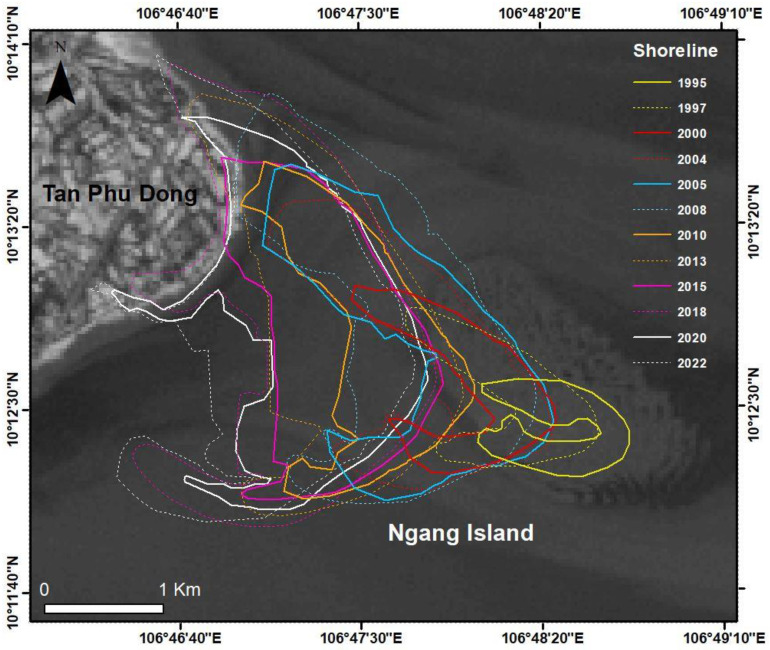
The morphological evolution of Ngang Island, Tien Giang Province, Vietnam, between 1995 and 2022.

Ngang Island evolved dramatically between 1995 and 2022, with its shoreline expanding steadily southwest between 1995 and 2022. The evolution commenced with fine-grained sediment accumulation in 1995 and compaction between 1995 and 2000, with a significant increase in vegetation cover and a decrease in non-vegetation cover including unconsolidated substratum (US) and compact substratum (CS) between 2000 and 2022 (Figure 3).

### 3.2. Mangrove Growth between 1995 and 2022

**Figure 4:**
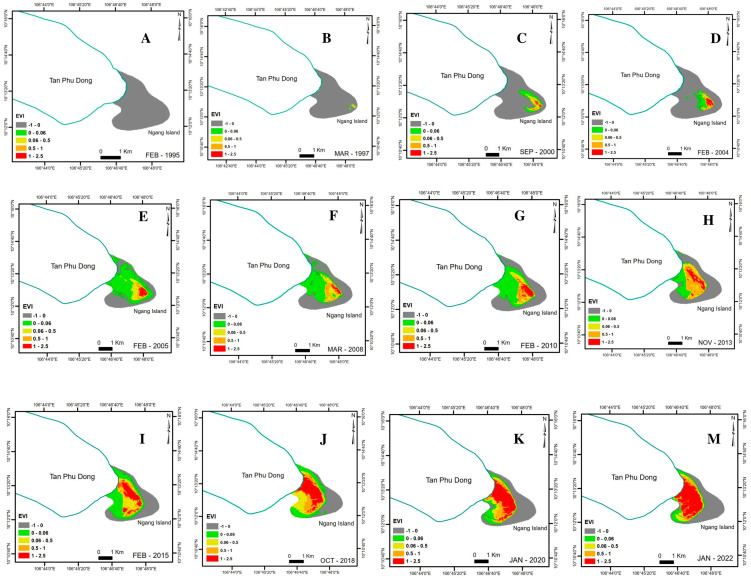
The morphological evolution and vegetation cover of Ngang Island, Tien Giang Province, Vietnam, between 1995 and 2022.

**Figure 5:**
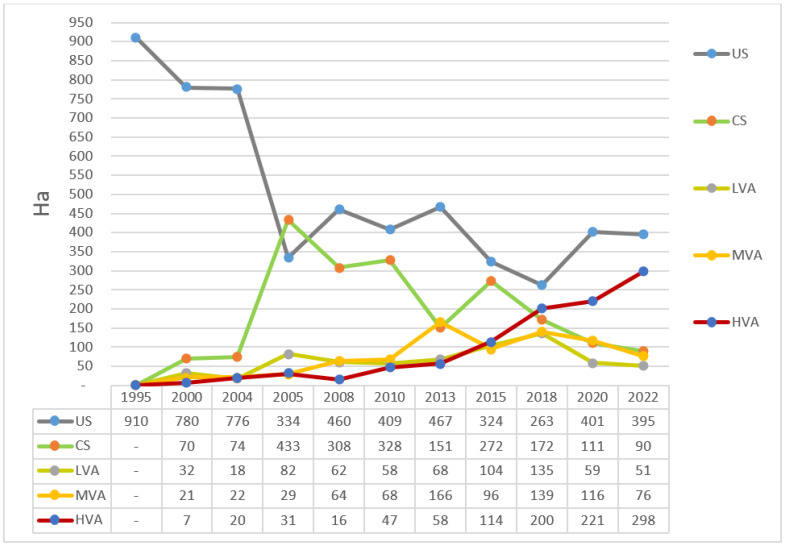
Vegetation cover changes at the study site between 1995 and 2022.

Ngang Island experienced a wide fluctuation in all vegetation categories between 1995 and 2022. The US decreased sharply from 910 ha in 1995 to 334 ha in 2005 and then fluctuated slightly from 409 ha in 2010 to 401 ha in 2020. The CS peaked at 433 ha in 2005 and then decreased steadily from 328 ha in 2010 to 111 ha in 2020. The low-vegetation area (LVA) reached 104 ha in 2015 and then decreased slightly to 59 ha in 2020. The medium-vegetation area (MVA) increased steadily from 21 ha in 2000 to 116 ha in 2020. The high-vegetation area (HVA) was the only one to increase steadily from 7 ha in 2000 to 221 ha in 2020. In 2022, there was a steady increase in HVA (from 221 ha in 2020 to 298 ha in 2022), although the date of the 2022 satellite retrieval was 28 January 2022 (Figure 4 and Figure 5).

**Figure 6:**
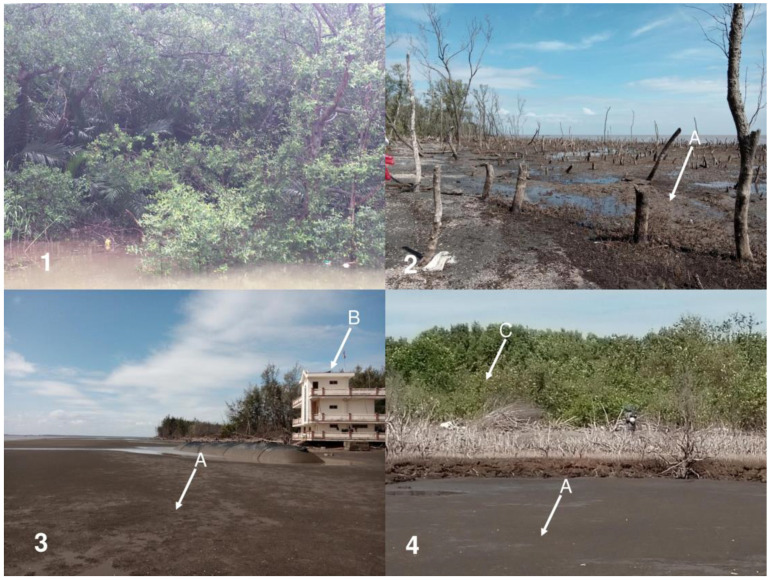
Status of the island in May 2023. (**1**) The mainland-facing section was rich in mangrove species (*Avicennia*, *Nypa*, *Bruigeria*, and *Sonneratia* species, MVA and HVA, 5th random monitoring site—see Figure 2A for further information). (**2**–**4**) Sea-facing sections. (**2**) The naturally regenerated *Avicennia* trees died because of the burial of their aerial roots in sand. The dried *Avicennia* trees were illegally chopped (LVC), 1st random monitoring site—see Figure 2A for further information). (**3**) Sand accumulation; (**4**), mature *Bruigeria* and *Avicennia* trees growing on elevated areas. (A) Sand found onshore in front of the Con Ngang Border Guard Station, (B) 2nd random monitoring site—see Figure 2A for further information). (C). The mangrove trees located at lower elevations died because of the burial of their aerial roots in sand, 3rd random monitoring site—see Figure 2A for further information).

The field visits to the random monitoring sites showed that the US was found mainly southwest of the island, located between Ngang Island and the Tan Phu Dong coast. While the US was still in liquid condition and inundated by the seasonal tidal regimes, the CS was found in areas where *Avicennia*, *Nypa*, *Bruigeria*, and *Sonneratia* species (MVAs and HVAs) grew strongly. The sea-facing area experienced a substantial death of mature mangrove *Avicennia* trees (LVA). The aerial roots of *Avicennia* trees were buried in sand, which killed the trees. The dead trees were illegally chopped up in the same year (Table 2 and Figure 6).

**Figure 7:**
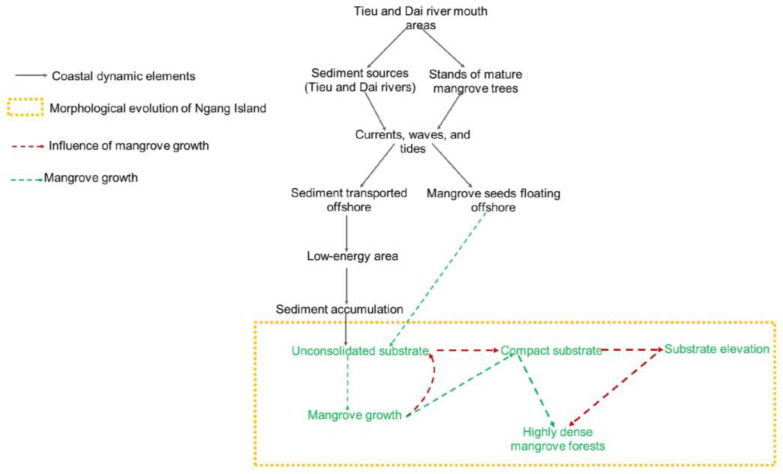
Summary of the spatiotemporal evolution of Ngang Island and the influence of mangrove growth.

The field observations showed that *Avicennia, nypa, Bruigeria,* and *Sonneratia* trees grew strongly along the mouths of the Tieu and Dai rivers. Mangrove seeds were seen floating around the island under the influence of seasonal tides and waves. The vegetated areas had a more compact surface and higher elevation than the remaining areas. The area located southwest of Ngang Island was less affected by strong incident waves and experienced a gradual accumulation of fine-grained sediment and mangrove growth, while the northeastern areas faced a robust sand accumulation and mangrove loss. The morphological evolution of Ngang Island and the influence of mangrove growth on the entire process are summarized in Figure 7.

## 4. Discussion

### 4.1. Morphological Evolution of Ngang Island and Mangrove Growth

The findings indicated that fine-grained sediment accumulated as US in the first stage of the morphological evolution of Ngang Island. The sediment accumulation that occurred was partly due to the area experiencing low-energy activities, as previously reported [60,66,67,68,69]. Sediment accumulation increased steadily and subsequently expanded the shoreline, as shown in Figure 3 and Figure 4. A previous study documented a similar compaction process [86]. The US provided favorable conditions for mangrove species to grow. Mangroves first grew in 1997 and formed a thin line off the Tan Phu Dong coast in 2000. The island experienced a slow natural growth toward the mainland between 2000 and 2010 and a significant growth between 2010 and 2022. The US compacted into CS over time due to the constant accumulation of fine-grained sediment and mangrove growth in the area, as shown in Figure 4. The landward mangrove growth established an appropriate protection of the area between Ngang Island and the Tan Phu Dong district coast, resulting in the area being sheltered over time. The more sheltered the area became, the more likely it was that the area would experience a strong mangrove growth. This helps explain why the MVA and HVA increased over time, while the US and CS decreased, as shown in Figure 4 and Figure 5. The areas without mangrove growth remained in liquid sediment conditions (US), while a strong mangrove growth elevated the substrate of the remaining areas, as shown in Table 2 and Figure 4, Figure 5 and Figure 6.

### 4.2. Sand Accumulation and Mangrove Death

Sand accumulation is a significant threat to the survival of mangroves on the island. Sand buried the aerial roots of mangrove trees (*Avicennia* and *Sonneratia* species) and killed the mangrove trees on sea-facing sites, as shown in Figure 6(2–4). This possibly helps to explain why the LVA decreased dramatically between 2015 and 2020, while sand accumulation was till dominant by 2020. The results of previous modelling indicated that sediments (coarse-grained—sand—and fine-grained—mud) were transported onshore by strong incident waves and subsequently relocated to other locations by longshore currents, which were created by strong incident waves in the Northeast season [62]. However, the origin of coarse-grained sediment—sand—has not been confirmed to date.

### 4.3. Implications of the Findings across a Wider Region

Mangrove forests grew naturally on CS, as shown in Figure 4, Figure 5 and Figure 6(1) and Table 2. The more mangrove trees grew, the more consolidated the substrate became, and the higher the EVI values became. Mangrove growth did not cause sediment accumulation in this study. These findings contradict the previous conclusion that mangrove trees promote sediment accumulation [61,62]. These findings are consistent with the conclusion that mangrove trees are likely to grow strongly in sheltered areas or areas that are not affected by physical and morphological pressures [43,44,45,46,47] and support previous findings concerning the capacity of mangrove trees to grow in intertidal mudflats [31,41,42,49].

Currently, the planting of mangrove plants has been prioritized by the existing coastal erosion control measures (for example, ecological engineering—mangrove planting—and engineered solutions for the construction of shoreline and offshore protection structures) to establish intertidal mudflats and stabilize eroded coastal areas in Southeast Asia [63,64,65], including Vietnam [48,66,67,68,69]. However, the opposite is true, as shown in Figure 3, Figure 4 and Figure 6(1) of this study. Thus, these findings suggest that the existing erosion control measures need to be updated or revise the primary goal of planting mangrove plants to accumulate fine-grained sediment into unconsolidated substrates as the first step toward stabilizing a site. Mangrove growth helps compact unconsolidated substrates as well as elevate compact substrates over time.

### 4.4. Limitations of the Study

In this study, the EVI values and the morphological evolution of Ngang Island were established through the analysis of satellite images and field observations to determine the natural growth of mangrove forests and the relationship between mangrove growth and the evolution of Ngang Island. However, neither soil samples nor soil profiles were collected and measured in this study, resulting in no information concerning the thickness of the accumulated sand, the sediment size, or the compacted substrate. If possible, additional modelling should be undertaken to gain insights into the origin of sand accumulation, the protection of mangrove forests from sand accumulation, and the spatiotemporal evolution of Ngang Island. Additional field measurements are needed to comprehend the layers and composition of the substrate in this site. These studies will provide a clearer explanation of what is reported here.

## 5. Conclusions

By investigating the relationship between mangrove growth and the morphological evolution of intertidal mudflats in the Tan Phu Dong district, Tien Giang, Vietnam, the authors found that fine-grained sediment accumulated as US as the first step of the morphological evolution of an intertidal mudflat, with sediment accumulation of 910 ha in 1995. The US provided favorable conditions for mangroves to grow, while mangrove growth helped compact the US into CS in addition to promoting continuous sediment accumulation, increased the vegetation cover of the island, and elevated the substrate density of the remaining areas. As a result, the US and CS decreased steadily between 1995 and 2020, from 910 ha in 1995 to 401 ha in 2020 and from 433 ha in 2005 to 111 ha in 2020, respectively. Meanwhile, the LVA, MVA, and HVA gradually increased between 1995 and 2015, from 0 ha in 1995 to 104 ha in 2015, from 0 ha in 1995 to 96 ha in 2015, and from 0 ha in 1995 to 114 ha in 2015, respectively. However, the LVA decreased slightly between 2015 and 2020 due to significant sand accumulation, which significantly killed the mangrove trees. In contrast, the MVA and HVA steadily increased between 2015 and 2020, from 96 ha in 2015 to 116 ha in 2020 and from 114 ha in 2015 to 221 ha in 2020, respectively. In 2022, there was a steady increase in HVA (298 ha in 2022), although the date of the 2022 satellite retrieval was 28 January 2022.

The findings of this study represent the first step towards understanding thoroughly the morphological evolution of Ngang Island through the development of shoreline changes and the calculation of the EVI values. Thus, there is no information concerning the thickness of the accumulated sand, the sediment size, or the compacted substrate. However, the results provide a technical reference for the revision or adjustment of the technical design of the existing coastal protection structures. Sediment accumulation should be emphasized as the first step in the morphological evolution of the examined intertidal mudflat, rather than mangrove growth. Similarly, further field work is needed to understand the origin of sand accumulation, the protection measures for mangrove forests, and the composition of the substrate.

## Figures and Tables

**Table 1:** Satellite images retrieved and analyzed in this study.

Year	Path	Row	Acquisition Date	Satellite Image	Cloud Cover	Cloud Cover Land	Spatial Resolution (m)
1995	125	053	14 February 1995	Landsat-5 (TM)	15	13	30
1997	125	053	18 March 1997	Landsat-5 (TM)	15	13	
2000	125	053	27 September 2000	Landsat-5 (TM)	15	13	
2004	125	053	11 February 2004	Landsat-5 (TM)	13	15	
2005	125	053	13 February 2005	Landsat-5 (TM)	12	12	
2008	125	053	9 March 2008	Landsat-5 (TM)	12	13	
2010	125	053	27 February 2010	Landsat-5 (TM)	20	23	
2013	125	053	18 November 2013	Landsat-8 (OLI)	18.34	17.63	
2015	125	053	9 February 2015	Landsat-8 (OLI)	2.86	3.37	
2018	125	053	31 October 2018	Landsat-8 (OLI)	6.80	6.42	
2020	125	053	6 January 2020	Landsat-8 (OLI)	2.28	2.18	
2022	125	053	28 January 2022	Landsat-8 (OLI)	2.28	2.18	

**Table 2:** Levels of vegetation cover, corresponding EVI values, and characteristics.

Levels of Vegetation Cover	Characteristics
US	Liquid sediment condition and seasonally inundated by local tidal regimes
CS	Compact substrate with scattered juvenile *Avicennia* trees growing
LVA	Scattered juvenile mangrove trees (*Avicennia alba*, *Bruiguiera gymnorhyza*, *Sonneratia alba*, and *Sonneratia caseolaris)* or areas with mangrove trees with roots buried in sand (dried mangrove trees)
MVA	Stands of growing *Avicennia alba*, *Bruiguiera gymnorhyza, Sonneratia alba*, and *Sonneratia caseolaris*
HVA	Stands of fully grown *Avicennia alba* and *Sonneratia alba*

## Data Availability

Data available in a publicly accessible repository.
